# Dietary Intake of Phytochemicals, Gut Microbiota, and Appetite Control

**DOI:** 10.3390/nu17162646

**Published:** 2025-08-15

**Authors:** Emad A. S. Al-Dujaili

**Affiliations:** Centre for Cardiovascular Science, Faculty of Medicine and Veterinary Medicine, Queen’s Medical Research Institute, University of Edinburgh, Edinburgh EH16 4TJ, UK; ealduja1@exseed.ed.ac.uk

## 1. Introduction

It has recently become apparent that plant phytochemicals can yield multiple benefits for human health, including optimizing physiological functions; modulating immune responses, inflammation, depression and anxiety, and the gut microbiota [1,2,3,4,5]; influencing cognitive function and Alzheimer disease [6]; enhancing stress adaptation; and impacting body system pathophysiology [7,8,9]. A prominent example is the probiotic and prebiotic activity of the major active constituent of phytochemicals, which modulate molecular pathways related to various body functions [10], including appetite control, which may manipulate the gut–brain axis to treat obesity [11,12], in addition to their antitumor and chemo-preventive activities [13,14]. Therefore, it is now recognized that gut health is essential for general health and wellbeing. The microbial content of the gut includes various colonies of beneficial bacteria and other microorganisms that have been found to influence the health status of the human body. Poor and fast-food diets and chronic stress cause constant gut inflammation-related compromised immunity and the development of various mood disorders through the gut–brain axis [9,12]. Thus, healthy, balanced, and gut friendly foods are important to maintain good health as well as minimizing stress [15]. Liu and colleagues [16] showed that dietary polyphenols exert various activities on aging hallmarks, including genomic instability, telomere attrition, epigenetic alterations, impaired macroautophagy, mitochondrial dysfunction, cellular senescence, altered intercellular communication, chronic inflammation, and dysbiosis [16].

At present, the growing interest in natural products is also driven by consumer demands, industry advancements, and the increasing prevalence of non-communicable diseases in aging populations [7,8,9]. As people seek safe and effective alternatives, the potential of natural products and functional foods in promoting health and wellness has gained momentum [7,17]. Functional foods and supplements rich in antioxidants polyphenols have been found to reduce the risk of chronic diseases, such as coronary heart disease, stroke, type 2 diabetes, obesity, and some cancers [18,19,20]. The pleiotropic effects of these polyphenols are evident regarding their role in redox modulation and inflammatory processes, molecular signaling, stem cell proliferation and differentiation, metabolism regulation, and hormonal imbalance, as well as their potential effects in cancer and neurodegenerative diseases [15,21]. The biological actions of diet and its active natural components have been mainly attributed to their multiple actions affecting various cellular and hormonal pathways [22]. For example, the mechanisms by which natural products could exert their antihypertensive effect have shown a multiplicity of actions (increased NO production, inhibition of renin release and ACE activity, angiotensin receptor and calcium channel blockade, antioxidant and anti-inflammatory activities, and opioid agonistic effects) [11,23,24].

## 2. Phytochemicals and Gut Microbiota Contributions from Reviews and Original Research

This Special Issue presents recent high-quality research in the field of appetite regulation, the gut microbiota, and the probiotic actions of phytochemicals, focusing on the investigation of gut-related mechanisms in relation to functional foods, including gut hormones, gastrointestinal motility, gut–brain communication, and the roles of diet and the microbiome. The authors and co-authors have published several articles on the subject, and some of their contributions have been listed among the list of contributions [1,2,3,4]. This Special Issue comprises the following five articles, including two research papers, two reviews, and one systematic review: One of the research papers investigated the effect of the short-term intake of elderberry juice rich in anthocyanin on fecal microbiota and reported an augmentation of the fecal microbiota and an improvement in glucose tolerance and fat oxidation [5]. The other research article is a clinical trial that compared the effects of high-protein versus high-carbohydrate diets on the gut microbiome, concluding that manipulating the impact of diet, microbiome, and metabolic health might require prolonged dietary intervention to generate a significant modification of the gut ecosystem to support diabetes T2 risk reduction [6]. Among the two reviews, one of them focused on postbiotics as adjuvant therapy in cancer care, concluding that postbiotics tend to have poor bioavailability. However, the use of symbiotics (a mixture of prebiotics and probiotics) would be expected to produce better homogeneous bioavailability [7]. The other review explored the significance of gut microbiota in diabetes pathogenesis and management, suggesting that interventions focusing on the gut microbiota of diabetic patients may have the potential as a promising approach to diabetes management [8]. The last article is a systematic review and meta-analysis exploring the effect of polyphenol supplementation on memory functioning in overweight and obese adults. It concluded that there was a potential positive effect of chronic supplementation with polyphenols on immediate retrieval in participants aged over 60 years, and that obesity is a risk factor for cognitive impairment [9].

## 3. Future Gut–Brain Axis Interactions

The gut–brain axis (GBA) is a bidirectional communication network between the gastrointestinal tract and the brain, which is modulated by gut microbiota and the related biomarkers. Malnutrition disrupts GBA homeostasis, exacerbating GBA dysfunction through gut dysbiosis, impaired neuroactive metabolite production, and systemic inflammation. Nutraceuticals, including probiotics, prebiotics, symbiotics, postbiotics, and paraprobiotics, offer a promising approach to improving GBA homeostasis by modulating the gut microbiota composition and related neuroactive metabolites [25]. A recent review aimed to elucidate the interplay between gut microbiota-derived biomarkers and GBA dysfunction in malnutrition, evaluating the potential of nutraceuticals in combating malnutrition [26]. Furthermore, it explored the future of personalized nutraceutical interventions tailored to individual genetic and microbiome profiles, providing a targeted approach to optimizing health outcomes. The integration of nutraceuticals into GBA health management could transform malnutrition treatment and improve cognitive and metabolic health.

## 4. Summary and Conclusions

In summary, understanding the importance of gut microbiomes has recently become vital for health, wellbeing, and disease. There has been considerable interest in research investigating the impact of gut microbiomes on metabolic diseases. The microbial content of the gut includes various colonies of beneficial bacteria and other microorganisms which have been found to determine the health status of the human body. Gut microbiota and phytochemical-rich diets can modulate the gut–brain axis, which plays a bidirectional homeostatic role between gut and brain functions. Dysbiosis and poor-quality diets cause a multitude of diseases, including cancer, neurodegenerative diseases, and non-communicable diseases, particularly in aging populations. Future and ongoing research studies should focus on the gut–brain axis and appetite control.
